# CT Utilization in a Level One Trauma Center in South Africa

**DOI:** 10.7759/cureus.29041

**Published:** 2022-09-11

**Authors:** Lara N Goldstein, Shabina Dawadi, Ilana M Viljoen

**Affiliations:** 1 Department of Emergency Medicine, HCA Florida Aventura Hospital, Aventura, USA; 2 Department of Diagnostic Radiology, University of the Witwatersrand, Johannesburg, ZAF

**Keywords:** trauma, imaging, emergency department, computed tomography, ct utilisation

## Abstract

Introduction

Computed tomography (CT) imaging forms an important component in the evaluation and management of patients with traumatic injuries. Many South African emergency departments (EDs) have a significant trauma-related workload, especially in the public sector, where there are limitations in resources relating to CT scanners. It is important to gauge the impact of traumatic injuries on CT utilization. The primary objectives were to quantify the number and type of CT imaging studies trauma patients received, as well as to determine the frequency of radiologically significant findings in a level one trauma center. The secondary objectives were to determine the CT utilization rate and describe the demographics of patients who received imaging.

Methods

This was a retrospective, quantitative, descriptive, cross-sectional study undertaken over two months at the level one trauma center of a tertiary, academic, public sector teaching hospital in Johannesburg, South Africa. The radiology department’s picture archiving and communication system (PACS) was used to evaluate the reports of trauma patients who were referred for a CT scan. The trauma center register was used to calculate the CT utilization rate.

Results

There were 5,058 trauma patients seen in the two months. A total of 1,277 CT scans were performed on 843 patients. CT brain accounted for 52% of all scans performed. Radiologically significant findings were demonstrated in 407 scans (354 patients), i.e. 31.9% of scans and 42% of patients. CT chest and peripheral angiogram demonstrated radiologically significant findings in 60.5% and 50.9% of scans respectively. Assault accounted for 55.8% of the injuries sustained and road traffic accidents accounted for 33.2%. The overall CT utilization rate was 16.7% i.e. 843 out of the 5,058 trauma patients underwent a CT scan.

Conclusions

South Africa has a substantial trauma burden which commonly necessitates CT utilization. It is concerning that blunt and penetrating assault continues to dominate these traumatic presentations. Worldwide, there is a broad range of described CT utilization rates and the findings at this level one trauma center fall within that range. ED clinicians are encouraged to continue carefully using this scarce resource in the trauma setting.

## Introduction

Trauma is a worldwide problem. It remains one of the foremost causes of death and disability [1]. In the Global Burden of Disease study from 2013, injuries accounted for 10.1% of the global burden [2]. The majority of injury-related deaths were from road injury, self-harm, falls, and interpersonal violence [1,2]. South Africa is no exception. Trauma-related mortality rates have previously been found to be six times the global rate and the road traffic accident injury rates were found to be double the global average [3].

Radiological imaging forms a crucial component in the management chain of trauma patients. Computed tomography (CT) scanning, in particular, has become a vital part of the diagnostic process. CT is a highly sophisticated resource which can quickly and effectively demonstrate multiple injuries in a trauma patient [4]. For this reason inter alia, CT utilization in the Emergency Department (ED) has increased significantly worldwide [5,6]. In the USA, usage increased by more than 300% from 1996 to 2007 with a more recent study in Taiwan showing an increase of 160% from 2009 to 2013 [5,6].

In South Africa, 45 million people utilize public sector healthcare services where there are 5 CT scanning machines available per one million population - it is a finite and costly resource [7-9]. This is dwarfed by developed countries such as Japan and the USA who have 111 and 42 CT scanners per million population, respectively [7-9]. A 2018 study from a level two trauma center in Johannesburg found that 36% of all CTs performed by the radiology department were referrals from the ED - traumatic injuries accounted for 57% of the usage [8]. It is important to ensure that a CT scanning service is utilized judiciously in the management of trauma patients especially in an income-restricted country. The result of the CT scan, whether positive or negative, ultimately needs to aid in the present clinical decision-making process and be weighed against the possible future risks of patient harm from the radiation [7].

The primary objectives of this study were to quantify the number and type of CT imaging studies trauma patients received at a level one trauma center, as well as to determine the frequency of radiologically significant findings. The secondary objectives were to determine the CT utilization rate and describe the patient demographics.

## Materials and methods

This was a retrospective, quantitative, descriptive, cross-sectional study undertaken over two months at the level one trauma center of a tertiary, academic, public sector teaching hospital in Johannesburg, South Africa.

All patients (adult and pediatric), with a history of trauma, who presented to the Chris Hani Baragwanath Academic Hospital level one trauma center during the study period, and who were referred for a CT scan, were included in the study. All the reports along with the necessary clinical information contained within them were available for review on the picture archiving and communication system (PACS). Ethics approval was obtained from the Human Research Ethics Committee of the University of the Witwatersrand (M191070). As data were collected retrospectively and patients had undergone the CT scans as per the standard of care, informed consent was not required from the individual patients.

All CT findings were considered radiologically significant except for skin lacerations, subcutaneous soft tissue injuries, chronic pathology, and any pathology not related to the mechanism of injury. The CT utilization rate was calculated by tallying the number of trauma patients who received a CT scan against the total number of patients seen in the trauma center during the study period. 

All data were captured electronically by a single abstractor in Microsoft® Excel (Microsoft Office 2019, Microsoft Corporation). Patient characteristics and CT results were evaluated using basic descriptive statistics. Analysis was performed using SAS (version 9.2 for Windows, SAS Institute, Cary NC, USA) and Microsoft® Excel (Microsoft Office 2019, Microsoft Corporation).

## Results

A total of 843 trauma patients were referred for CT scans and included in the study. There were 44 reports excluded from evaluation due to missing information. Over the study period, 5058 patients were seen in the level one trauma center. The CT utilization rate was therefore 16.7% (843/5,058). 

Males comprised 80.7% and females 18.5% of the study population. Sex was not documented in 0.8% of patients. 

The median age was 32 years (interquartile range [IQR] 25 - 41). Patients older than 65 years comprised 3.2% of the population and patients younger than 18 years comprised 7.6% of the study population. Age was not documented in 10.9% of patients. 

The total number of CT scans performed was 1,277, the breakdown of which is presented in Table 1. Out of the 1,277 CT scans, 407 (31.9%) demonstrated radiologically significant findings. This was in 354 patients (42.0%).

**Table 1:** Breakdown of total CT scans performed.

Scan type	n (%)
Brain	664 (52.0)
Cervical spine	274 (21.5)
Abdomen/pelvis	110 (8.6)
Chest	86 (6.7)
Peripheral angiogram	57 (4.4)
Neck angiogram	52 (4.1)
Whole spine	28 (2.2)
Miscellaneous	6 (0.5)

A whole-body CT “pan scan” (which included a non-contrast CT brain, CT cervical spine (C-spine) as well as contrast-enhanced CTs of the chest, abdomen, and pelvis) was performed in 6.5% of patients.

**Figure 1:**
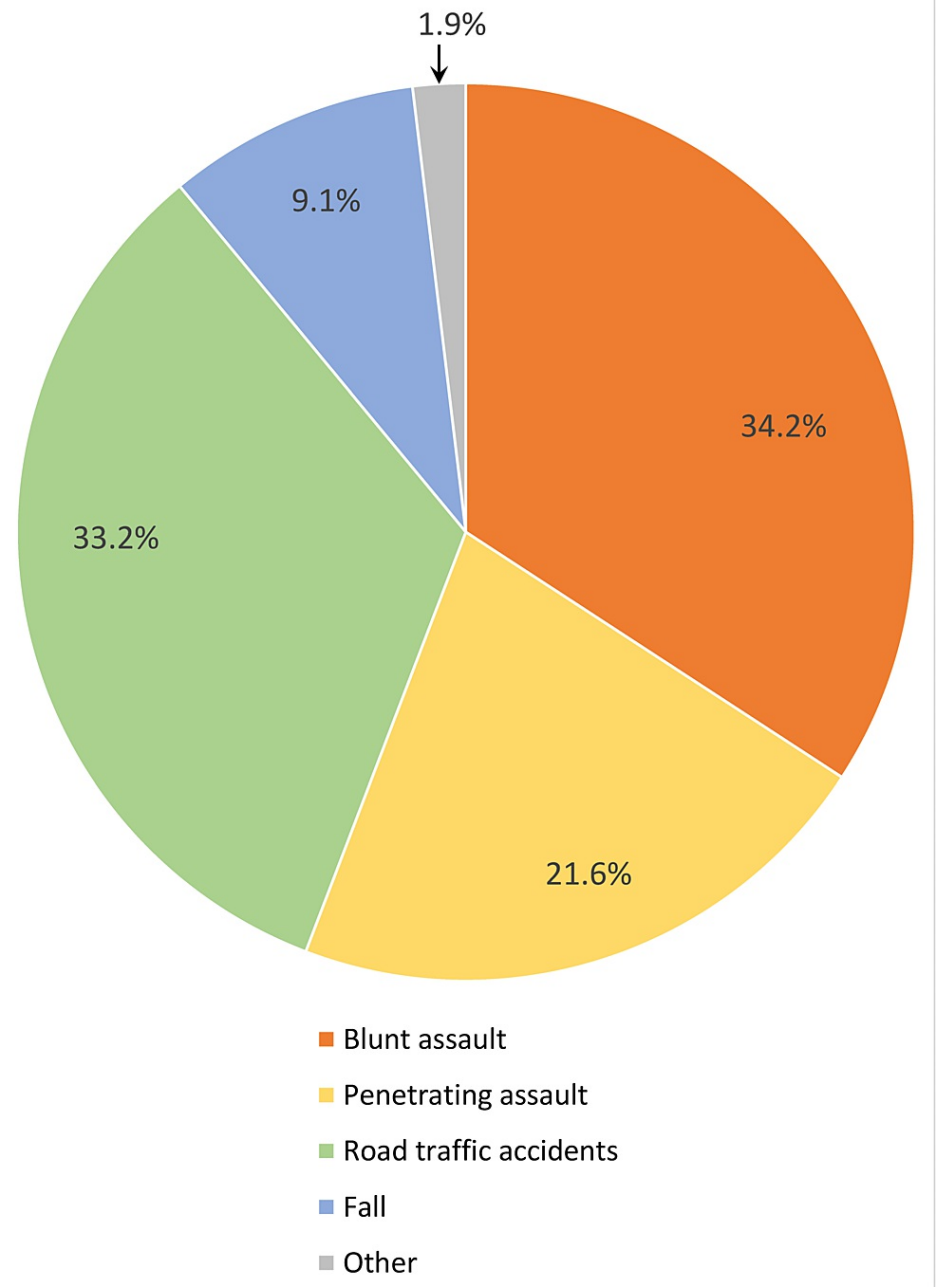
Mechanism of injury.

**Figure 2:**
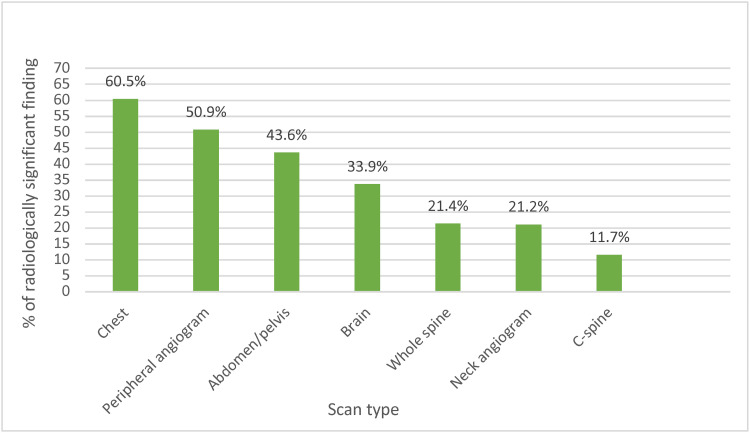
Percentage of scans with radiologically significant findings.

Figure 1 demonstrates the mechanism of injury of the patients. Blunt trauma accounted for 76.5% of injuries. Figure 2 demonstrates a breakdown of each type of CT scan with radiologically significant findings. 

Table 2 shows the frequency of the commonest injuries for each CT scan type.

**Table 2:** Frequency of injuries per scan type in decreasing order. Percentages displayed are per scan type i.e. 32.4% of CT brain scans demonstrated a skull fracture.

Scan type	Injury (%)
Brain	Skull fracture (32.4)
	Subarachnoid hemorrhage (30.2)
	Contusion (30.2)
	Facial fracture (24.4)
	Orbit injury (23.1)
Cervical spine	C7 fracture (37.5)
	C6 fracture (31.2)
Chest	Rib fracture (63.4)
	Pulmonary contusion (48.0)
	Hemopneumothorax (36.5)
Abdomen/pelvis	Liver injury (29.1)
	Pneumoperitoneum (22.9)
	Pelvis fracture (22.9)
	Renal injury (14.5)
	Spleen injury (12.5)
	Hemoperitoneum (12.5)
Peripheral angiogram	Brachial artery injury (63.6)
	Superficial femoral artery injury (11.7)
Neck angiogram	Vertebral artery injury (18.1)

## Discussion

The use of CT scanning in medicine has increased dramatically over the last few decades. This increase is happening to a greater extent in the ED, the frontline of the hospital [5]. The global burden of injury is known to be inversely proportional to income which makes low- and middle-income countries particularly vulnerable to a large volume of trauma; South Africa is one such example of this [2,3,7,10]. Unfortunately, the public sector hospitals in South Africa have a low CT scanner-to-patient ratio making it a limited resource that needs to be utilized wisely [7,9].

In this study, there were 4.3 times more males than females who sustained trauma that required CT imaging. This is consistent with other study populations of traumatic injuries [8,11,12]. The median age of patients in this study also mirrored the trend previously noted in South Africa, with a younger patient population presenting to the ED with traumatic injuries [13]. Of note, South Africa is atypical when comparing the mechanism of injury patterns to worldwide trends [3]. Elsewhere, road traffic accidents and other injuries predominate. However, as in this study, assault was the mechanism of injury in more than half of the patients [3,7]. Interpersonal violence is globally recognized as an important public health issue, with low- and middle-income countries being particularly afflicted [12].

The ordering of whole-body CT pan scans as well as multiple CT scans being ordered per patient resulted in there being more scans than patients. A patient could have had more than one anatomic region scanned depending on their mechanism of injury, clinical presentation, and imaging requirements. It is often debated whether to use whole-body CT pan scan or selective CT scanning in the management of trauma patients [4]. A pan scan is particularly helpful in the setting of blunt polytrauma. It can quickly localize multiple injuries and aid in triaging the management of patients [4,14]. It can guide the conservative management of patients with solid organ injuries and help to differentiate patients who need intensive care or surgical intervention [14]. Whole-body pan scans demonstrated clinical utility in the vast majority of patients in a level one trauma center study from Pietermaritzburg, South Africa [14]. 

CT of the brain was the most commonly ordered investigation overall. This is similar to findings from previous studies in South Africa conducted in Johannesburg and Cape Town [8,15]. In the Cape Town study, 90% of the assault victims required a CT brain [15]. When head trauma is sustained, it can have devastating consequences. Imaging is often necessary to evaluate for traumatic brain injury which may require neurosurgical intervention [16].

Our study found that C-spine CT scans had a lower positive yield compared to CTs of the brain. This might be because C-spine CTs are commonly coupled together with CT brain orders due to the known increased risk of C-spine injuries in head trauma [17]. Kulvatunyou et al. noted a positive yield of 0.7% if a CT cervical spine was done for isolated, direct blunt head trauma [18]. Although it is a low-yield investigation, it will likely continue to be ordered in conjunction with a CT brain. The patient-related morbidity and mortality costs as well as the potential litigation expenses from a missed cervical spine injury are just too high.

Neck and peripheral CT angiography are effective imaging modalities to evaluate for vascular trauma [19,20]. In a prior South African study, the most common indication for performing peripheral CT angiography was gunshot wounds [21]. This was also the major mechanism of injury contributing toward peripheral CT angiography being performed in the current study and may account for the more significant number of injuries noted in this group. 

Significant imaging findings were noted in less than half of the patients in this study. A positive yield rate of 53.8% was found in a study that was conducted previously in a nearby hospital in Johannesburg, South Africa [8]. This discrepancy may be related to our study being conducted at a level one trauma center compared to the other study which was at a level two trauma center. This highlights the principle that a lower positive yield is likely related to increased CT utilization which was potentially the case in our level one trauma center [8]. 

There are variable ED CT utilization rates throughout the world. These range widely between 2.8% to 33% [6]. A direct local comparison could not be made as, to the best of our knowledge, local level one trauma center utilization rates have not previously been described. A level two trauma center in Johannesburg showed a CT utilization rate of only 4.6%. This finding however was for both traumatic and non-traumatic conditions [8]. Factors that may contribute to increased CT utilization in the ED include, but are not limited to, the ease of access to radiology services, utilization to increase patient throughput, and the non-invasive advantage of CT compared to other traditional, more invasive methods such as peritoneal lavage [22]. Many ways to improve positive yields have been proposed including using clinically-based guidelines [23]. Although there is a strong argument for decreasing CT usage, it is important to note that there are also benefits to current utilization patterns. A study conducted by Salim et al. at a level one trauma center in Los Angeles, USA, found that of patients without obvious external signs of injury who underwent a whole-body CT pan scan, 18.9% had a change in management based on abnormal CT findings which were not suspected clinically [24]. 

However, increasing CT utilization needs to be weighed against cost [5]. In a study conducted in Bloemfontein, South Africa, CT scan costs accounted for 83.8% of the total radiological expenditure for violence-related injuries while in the prior fiscal year, the Department of Health showed the second highest expenditure of all government departments [25]. These financial implications represent only a single facet - the direct cost - of a multi-faceted process. This expenditure needs to be balanced against the costs of missing clinically significant injuries in trauma patients which can occur in 15 to 22.3% of patients [26]. This can further be counterbalanced by the potential cost-saving of the decreased likelihood of hospitalization for patients who undergo CT imaging [27]. The stochastic effects of radiation exposure from CT also need to be taken into consideration [28]. Although the benefits of CT imaging for traumatic injuries generally outweigh the risks of carcinogenesis, it is important to acknowledge the associated health risks [29]. Trauma patients are more likely to receive radiation exposure in general as well as receive higher radiation doses than other hospital-based populations [29].

This study is strengthened by the size of the study population as well as it having been conducted in a level one trauma center. Limitations include the fact that data were collected retrospectively and only over two months, which could have resulted in seasonal variations of traumatic presentations being missed. Furthermore, it is difficult to extrapolate findings from only one facility suggesting the need for trauma registry-based imaging research. This would preferably be from a country-wide database from all hospitals and not just level one trauma centers. The lack of electronic medical records for the CT scan report documentation as well as the registration records may mean that some data could have been inadvertently omitted.

## Conclusions

South Africa has a substantial trauma burden which commonly necessitates CT utilization. It is concerning that blunt and penetrating assault continues to dominate these traumatic presentations. Worldwide, there is a broad range of described CT utilization rates and the findings at this level one trauma center fall within that range. With the large number of trauma patients in South Africa needing CT imaging, referring clinicians need to remain aware of the risks, benefits, costs, and impact thereof.
